# Effects of filler on the microstructure and corrosion of similar and dissimilar gas inert tungsten arc welding aluminum alloys joints

**DOI:** 10.1038/s41598-023-44421-y

**Published:** 2023-11-03

**Authors:** Elshafey Ahmed Gadallah, Mohamed Ibrahim Abd El Aal, Abdelkarim Yousif Mohamed, Hossam Hemdan El-Fahhar

**Affiliations:** 1 Mechanical Production Department, Faculty of Technology and Education, Suez University, Suez, 43527 Egypt; 2 Mechanical Engineering Department, College of Engineering at Wadi Addawaser, Prince Sattam Bin Abdulaziz University, 18734 Wadi Addawaser, Saudi Arabia; 3 Mechanical Design and Production Department, Faculty of Engineering, Zagazig University, Zagazig, 44519 Egypt

**Keywords:** Mechanical engineering, Mechanical properties, Mechanical properties

## Abstract

Welding of dissimilar aluminum alloys has been widely used in many industrial applications. However, the selection of filler type still attracts significant interest in the welding research area. The present work concerns the effect of filler metal on the microstructure and corrosion of weld joints of dissimilar aluminum alloys. AA 5083 and AA 6082 alloys were welded by tungsten inert gas welding (GTAW) using filler metals ER 4043 and ER 5356. The microstructure observations and the corrosion test of the weld joints were carried out. Solidification cracks were observed in the ER 4043 weld zone, whereas defect-free joints were obtained using a mix filler welding process. A galvanic corrosion was observed on the boundary between the filler rod ER 4043 weld zone and AA 5083 base alloy. From the corrosion standpoint of view, the using of ER 4043 electrodes is not preferred for welding 5000 series aluminum alloys, whereas ER 5356 filler electrode is more favorable than ER 4043 filler electrode either for dissimilar welding of AA 5083 and AA 6082 alloys or individual welding of both aluminum alloys. No galvanic corrosion is observed between ER 4043 fillers and AA 6082 base alloy.

## Introduction

It is well known that Al alloys' fusion welding has different defects. The welding of the Al alloys defects includes pores, loss of particular elements, hot cracking, stress corrosion cracking, and mismatch between filler alloy and workpiece material; those reduce the weld strength^1–3^. The electrode selection during Al alloys' fusion welding affects the welding joint's properties and quality. Improper selection of welding electrodes affects aluminum alloys' corrosion resistance when used in marine or harsh environments. AA 5083 and AA 6082 represent two different families of Al alloys, namely the strained hardening alloys (AA 5083) and the heat-treatable alloys (AA 6082), which have good mechanical properties, corrosion resistance, and well workability and Weldability^4–6^.

The welding of aluminum alloys considered a difficult process due to its high thermal and electrical conductivity, thermal expansion coefficient, and refractory aluminum oxide (Al_2_O_3_)^7^. So, aluminum alloys gain thier strength through strain hardening, that missed during welding due to the recrystallization of grains. Moreover, the softening of the partially melted zone (PMZ) and the heat-affected zone (HAZ) and so grain growth in the fusion zone occurs^8–11^. However, the welded Al alloys strength and corrosion resistance can improved by controlling the size and distribution of intermetallics, such as Mg_2_Si, Al_6_Mn, and Al_6_ (Mn, Fe)^10,11^. Recent research has shown that incorporating nanomaterials, such as CNTs, TiO_2_, and Al_2_O_3_, in GTAW welding of Al alloys can improve the microstructure and mechanical properties while reducing welding defects^12,13^. Kumar. P. et al. and other researchers have reported that changes in welding parameters, such as welding current, speed, and heat input, can also affect the microstructure by altering the grain size and distribution of precipitates in the HAZ. Mustafa. U. et al. study the mechanical and corrosion properties of GTAW of aluminum alloys^14^.

Microstructures with fine grains at the weld zone was detected at the interfaces of ER 4043 filler wire in AA 6082 and AA 5083 alloys in different joints^15^. The fine grain structures observed in various areas contribute to increase the tensile strength of ER 4043 sealant welds^15^.

Welding AA 5083 and AA 6082 alloys attracted different previous works^16,17^. Bo Wang et al. modifies the ER 4043 filler electrode by adding Ti and Sr to improve the mechanical properties of AA 6082 weldments using GTAW^16^. They concluded that the addition of the Ti and Sr together improves the welded zone's microstructure and leads to the enhancement of mechanical properties. Moreover, Mohd Noor C. W. et al.^17^ studied the effect of the welding by GTAW and inert metal gas (MIG) welding on the microstructure and mechanical properties of AA 5083 welded joints used in shipbuilding. They found that fine microstructure was obtained for welded joints using GTAW, whereas coarse microstructure for welded joints used MIG. Also, the GTAW process gives welded joints high ultimate tensile strength and ductility compared with the MIG process.

In many cases, dissimilar welding of aluminum alloys becomes necessary. In this case, the problem of filler selection becomes more complicated due to the macrosegregation results from the inherent compositional variations between the filler and base metals^18–20^. Moreover, Aendraa Azhar Abdul Aziz et al.^21^ investigate the effect of different filler alloys on the mechanical properties and microstructure of welded AA 6061 aluminum alloy using MIG by two different fillers, ER 4043 and ER 5356. Aendraa Azhar Abdul Aziz et al. concluded that the amount of Si and Mg in the weld zone plays a vital role in controlling the microstructure and mechanical properties of the welded joints^21^. Che Lah et al.^22^ also studied the effect of fillers ER 4043 and ER 5356 on the porosity distribution of AA 6061 welded joints. The presence of Si and Mg in the weld zone was observed to affect the porosity distribution. However, no significant effect of the Si and Mg addition was observed on the hardness profile for both filler materials.

Aluminum and its alloys have numerous advantages, such as reducing structural weight and hull maintenance^23^. The 5xxx and 6xxx series alloys are widely accepted materials for shipbuilding due to their sufficient strength, good corrosion resistance, and ability to withstand corrosive atmospheres. These alloys used in construction of hull structures, superstructures, and decks of ships^24^. The alloying element of the 5xxx series include Mg, which provides good strength and outstanding corrosion resistance and toughness^25^. The 6xxx series alloys with the binary aluminum-magnesium silicide system (Al-Mg_2_Si) provides almost equal strength with slightly lower corrosion resistance than the 5xxxx series alloys^25,26^. These alloys commonly used for the construction of deck panels and marine frames of ships.

According to the problems noted while welding different alloys with different compositions using different electrodes^27,28^. The need for further investigations to overcome those problems is still needed. So, the current research aims to investigate the effect of using two welding electrodes, ER 4043 and ER 5356, simultaneously and individually for welding AA 6082 and AA 5083 using GTAW on the microstructure and corrosion resistance of the welded joints.

The present work will be undertaken to reach the following aims:

1. Reach a proper selection of filler electrodes in welding similar and dissimilar AA 6082 and AA 5083 weld joints.
2. Investigate the effect of the filler electrode material and base metal on the welding microstructure defects.
3. Study the effect of single and multiple fillers in the weld zone on the corrosion resistance of similar and dissimilar AA 6082 and AA 5083 weld joints.

## Materials and methods

The materials used in this research were wrought aluminum alloys 5083-H111 (AA 5083) and 6082-T6 (AA 6082) plates with a thickness, width, and length of 6 × 200 × 300 mm^3^. The chemical composition shown in Table 1 indicates that the AA 5083 and AA 6082 belong to the 5XXX and 6XXX series aluminum base alloys. The Mg and Si considered the main elements with high content after Al in the AA 5083 and AA 6082. The presence of the Mg in AA 5083 improves its strength, corrosion resistance, and weldability. Moreover, Si reduces melting temperature and improves fluidity.

**Table 1:** Chemical composition of materials (wt.%).

Material	Si	Fe	Cu	Mn	Mg	Cr	Zn	Ti	Al
AA 5083-H111	0.35	0.38	0.08	0.6	4.72	0.09	0.1	0.08	Bal
AA 6082-T6	1.01	0.22	0.02	0.56	1.02	0.13	0.03	0.03	Bal
ER 4043	5.25	0.8	0.3	0.05	0.05	0.0	0.1	0.2	Bal
ER 5356	0.25	0.4	0.1	0.14	4.80	0.12	0.09	0.1	Bal

Silicon alone in aluminum produces a non-heat-treatable alloy; however, in combination with Mg, turrn it into a precipitation-hardening heat-treatable alloys. Furthermore, both alloys contain reasonable amounts of Mn and Fe, those enhance the strength and significantly improve low-cycle fatigue resistance.

**Figure 1:**
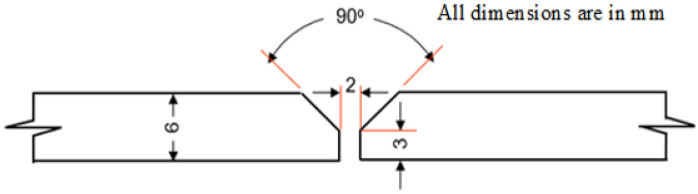
Weld joint preparation.

The Al plate edges prepared to have V-groove butt joined for using GTAW welding, as shown in Fig. 1, according to the American Welding Society (AWS) code D1.2/D1.2M^27,29^. ER 4043 and ER 5356 filler metals with chemical composition given in Table 1 are used in the current study. ER 4043 filler is suitable for welding heat-treatable base alloys, especially the 6XXX series alloys. It has a lower melting point and more fluidity than the 5XXX series filler alloys, and is preferred by welders because of its favorable operating characteristics. ER 4043 filler wires have a lower weld cracking sensitivity for 6XXX aluminum grades, which is a better option for welding than the ER 5356 filler wire. ER 4043 weldments can be used for service temperatures up to 65 °C (150 °F).

On the other hand, ER 5356 has become the most commonly filler during the MIG and GTAW of the aluminum alloys because of its good strength and feed-ability. It is designed to weld Al 5xxx and 6xxx series. However, ER 5356 Filler not suitable for service temperatures exceeding 150 °F (65 °C). The formation of Al_2_Mg at elevated temperatures at the grain boundaries makes the alloys prone to stress corrosion.

Although ER 4043 and ER 5356 fillers have many advantages, they suffer from many defects. Considering the difference in advantages and disadvantages of the two fillers besides the difference in their cost. The present work considers a further comparison between them and introduces a new approach to investigate the effect of using a mixture of them.

**Figure 2:**
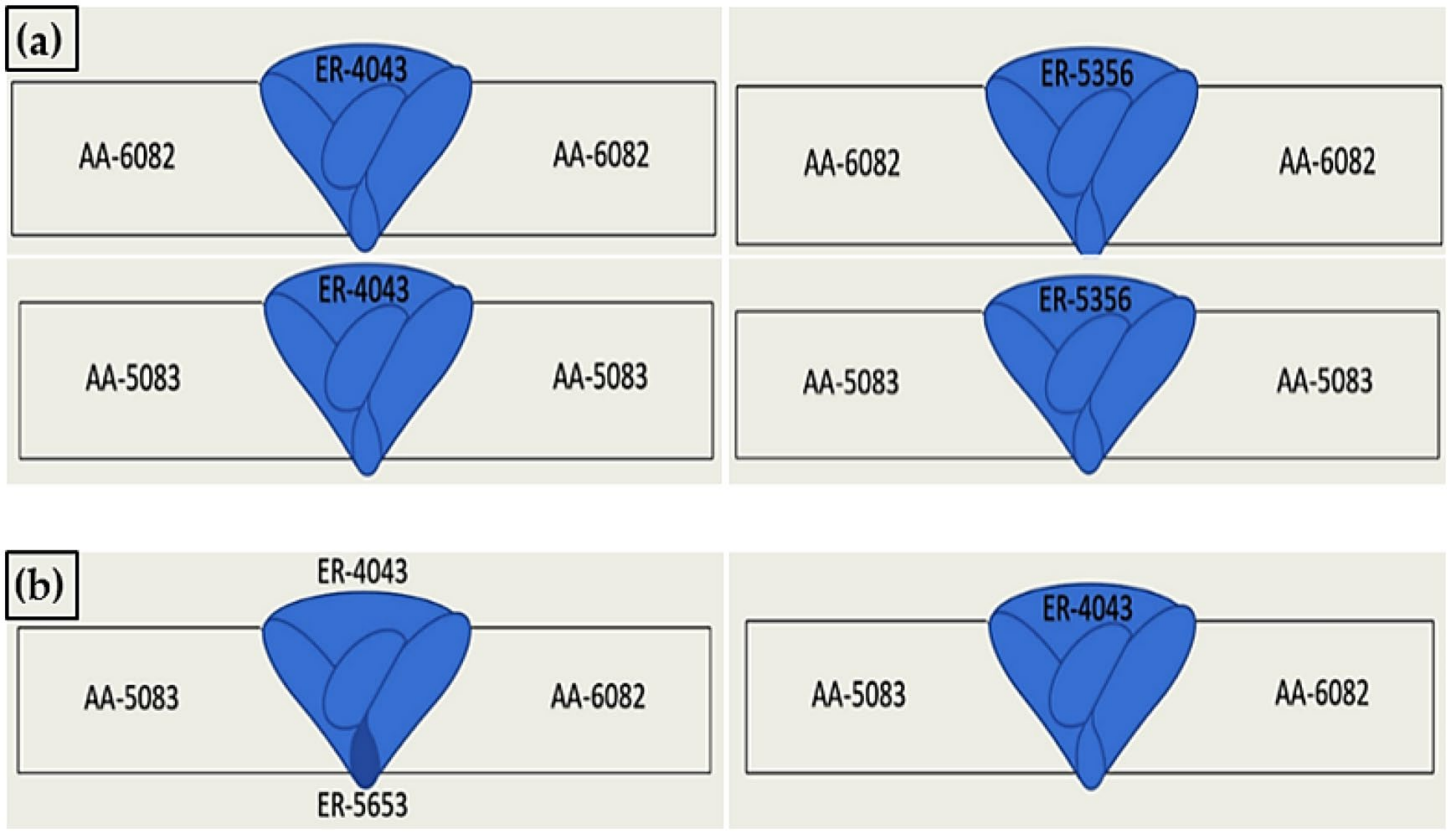
Schematic illustrations of similar and dissimilar joints: (**a**) similar and (**b**) dissimilar joints specimens.

The weld joints were cleaned to eliminate the rust, dust, and oil that may penetrate the weld zone, causing a weld defect. GTAW process was performed at the CEANDRO Shipyard Company for Ship Building and Repair, Suez, Egypt, using a Lorch Saprom S3 Mobil welding machine. The GTAW carried out the welding process for the similar and dissimilar welding joints, using filler metals with 1.2 mm diameter ER 4043 and ER 5356. Pure argon was used to protect the weldments surface from oxidation. Figure 2 shows the schematic diagram of similar and dissimilar weld joints of AA 5083 and AA 6082 aluminum alloys. The GTAW welding parameters, minimum and maximum heat input values are given in Table 2.

**Table 2:** GTAW welding parameters and heat input.

Size of filler (mm)	Current (A)	Voltage (V)	Travel speed (mm/min)	Torch distance (mm)	Heat input (KJ/mm)
1.2	130	21.8	250—350	15	Min. 0.49 Max. 0.68

The microstructure observations of the different weldments performed using both Olympus optical microscope (OM) model (BX41M-LED, Center Valley, PA, USA) and the scanning electron microscope (SEM) model FEI INSPECT-S50 (FEI, Austin, TX, USA). The specimens were ground and polished according to standard metallographic practice ASTM E3-11^30^. Keller’s reagent (3 ml HCl, 5 ml HNO_3_, 2 ml HF, and 190 ml distilled H_2_O) was used as an etchant^31^. Dispersive X-ray spectroscopy (EDS) area analysis also performed using the same SEM.

**Figure 3:**
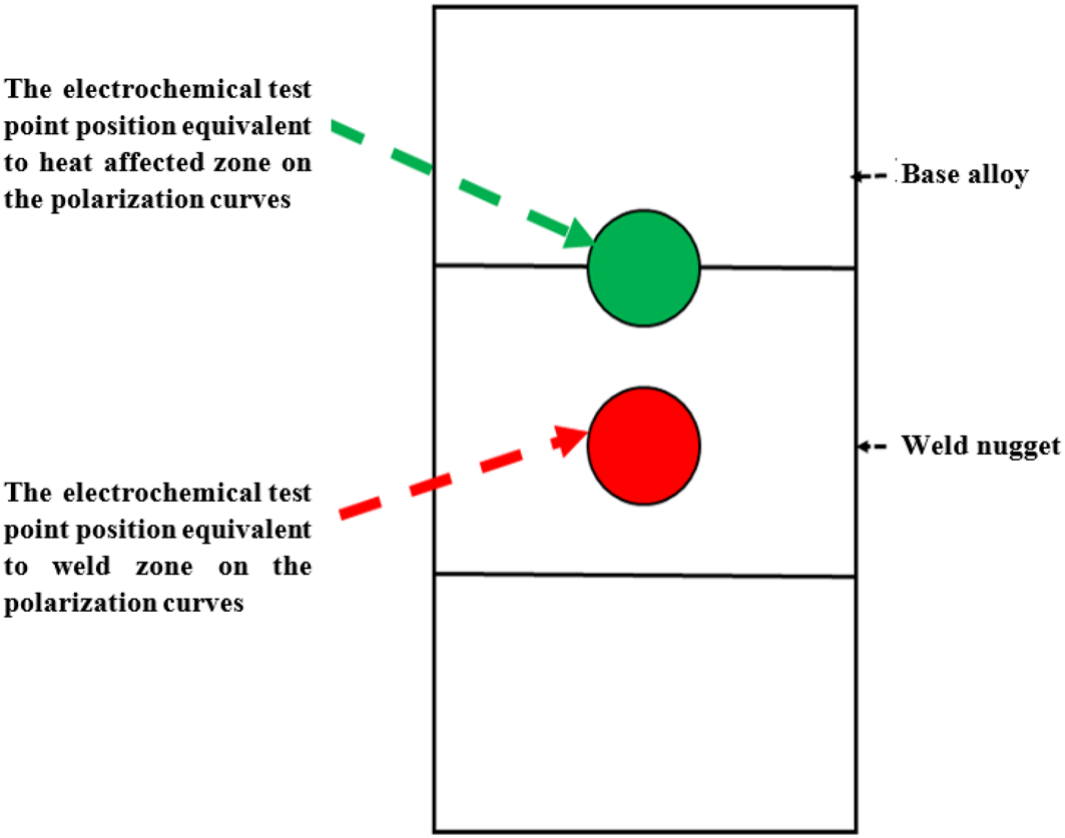
Schematic illustrations of the position of the corrosion test.

**Figure 4:**
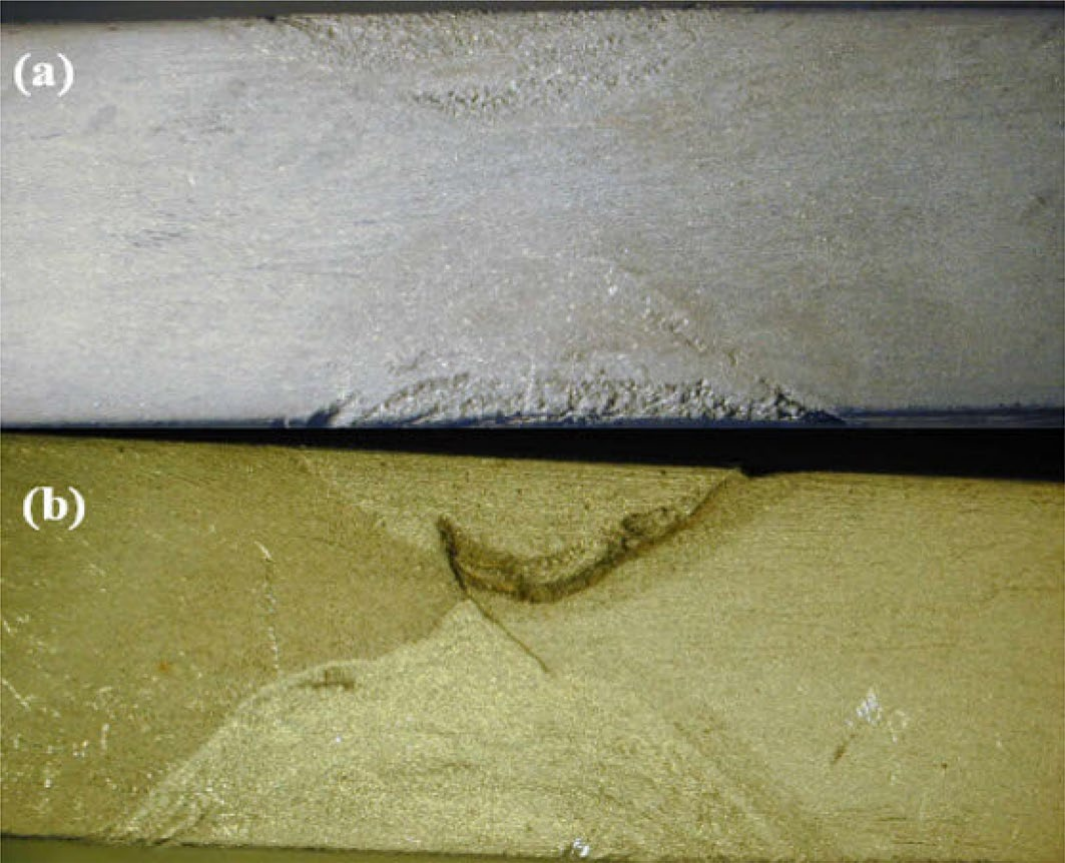
Specimens: (**a**) before and (**b**) after the corrosion test.

The welded joints were cut in the transverse direction of welds into specimens with dimensions of 60 × 15 × 6 mm^3^ according to standard metallographic practice ASTM E3-11^30^. The corrosion specimens were prepared in the same way used for the microstructure one according to ASTM E3-11^30^. Electrochemical polarization tests were performed in a corrosion cell containing 250 ml 3.5 wt%. NaCl solutions at room temperature (RT)^32,33^. A scan rate of 0.5 mV s^−1^ was applied. Electrochemical parameters were measured using a potentiostat AUTOLAB^®^ PGSTATE30. Platinum gauze was used as a counter electrode, and silver/silver chloride (Ag/AgCl) was the reference. The position of the corrosion test is indicated in Fig. 3. The images of the corrosion samples before and after the corrosion test shown in Fig. 4.

## Results and discussion

### Microstructure observations

#### Welding of similar AA 6082 and AA5083 alloys joints

##### Similar welding joints using ER 4043 filler

**Figure 5:**
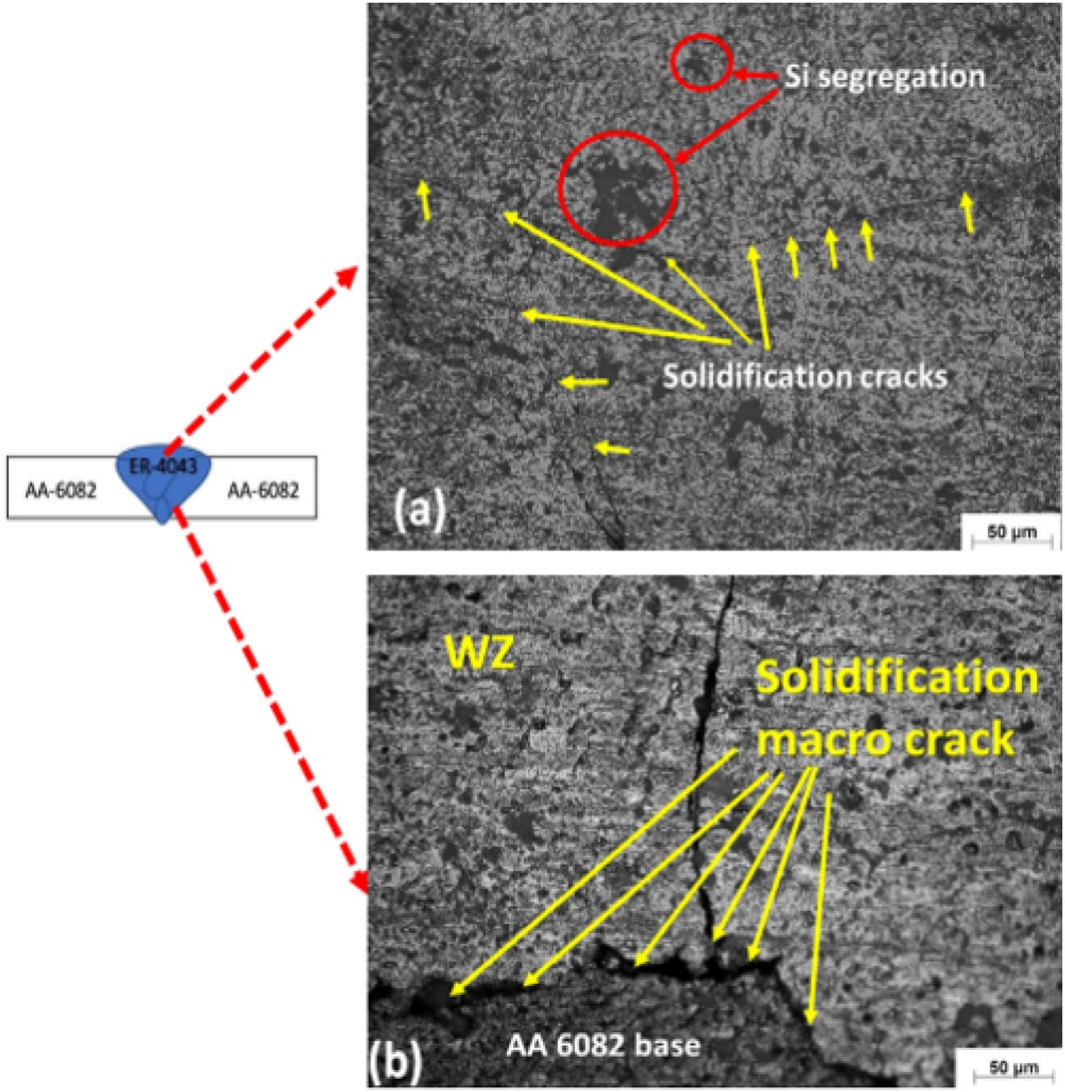
Optical microscopy photomicrographs of the weld zone of AA 6082 base alloys welded joints ER 4043 filler metal showing: (**a**) the weld zone reveals two types of defects they are solidification cracks (yellow arrows) and silicon segregation (red circles) and (**b**) the welded joints-heat affected zone interface at the root of AA 6082 base alloy welded joints using ER 4043 filler.

The microstructure of the similar AA 6082 welded joints using ER 4043 filler was examined in the welded zone (WZ) and heat-affected zone (HAZ). The micrographs show apparent solidification cracks in the WZ and WZ-HAZ root interface of the AA 6082 joints welded with ER 4043 electrode, as shown in Fig. 5a, b. Si particle segregation was also observed in the WZ, as noted in Fig. 5a.

**Figure 6:**
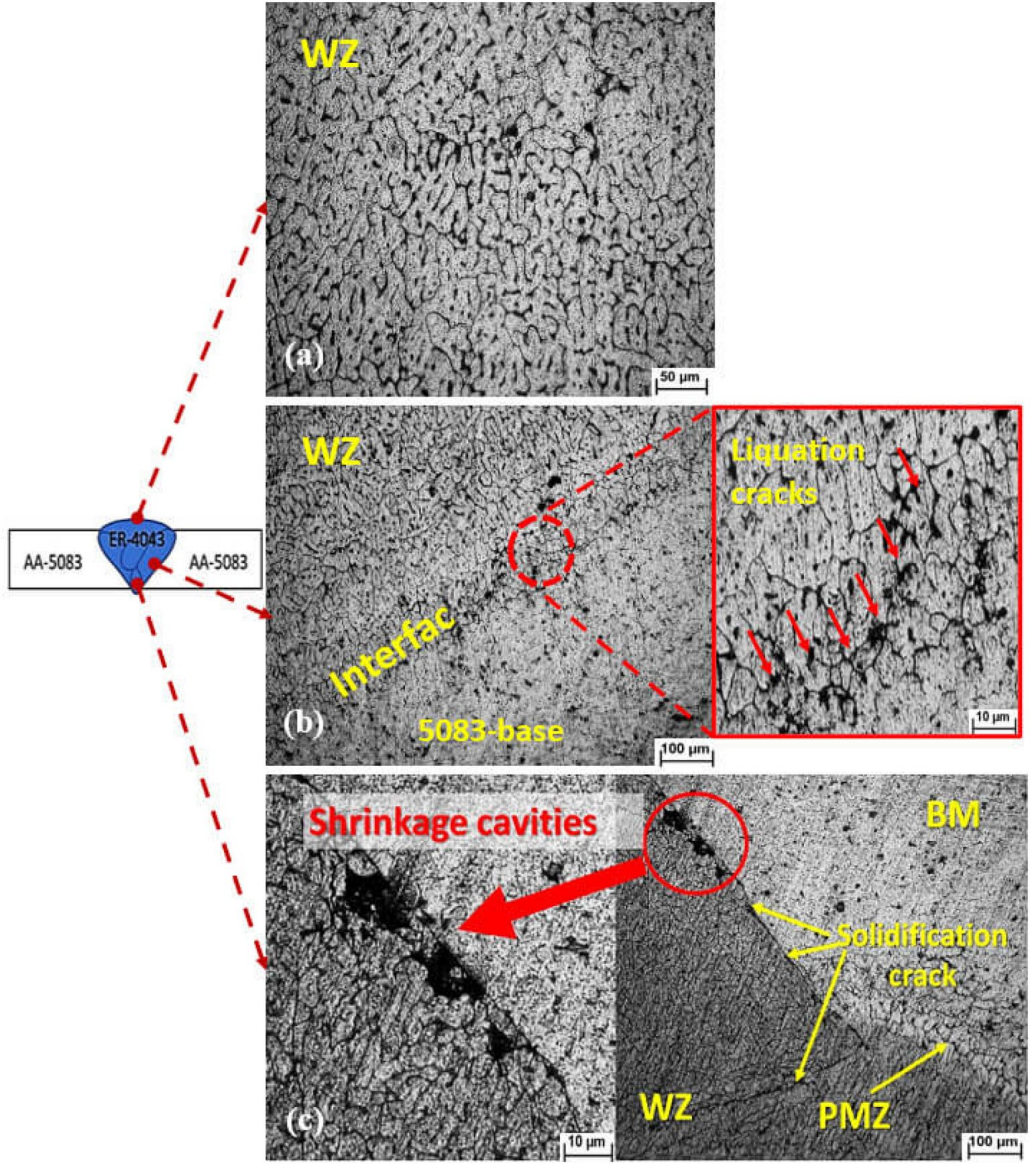
Optical microscopy photomicrographs of the weld zone of AA5083 base alloys welded using ER 4043 filler: (**a**) the weld zone reveals micro shrinkage at grain boundaries, (**b**) the welded joints-heat affected zone interface of AA 5083 base alloy welded joints and (**c**) the interface of WZ-HAZ at the root of the joint.

**Figure 7:**
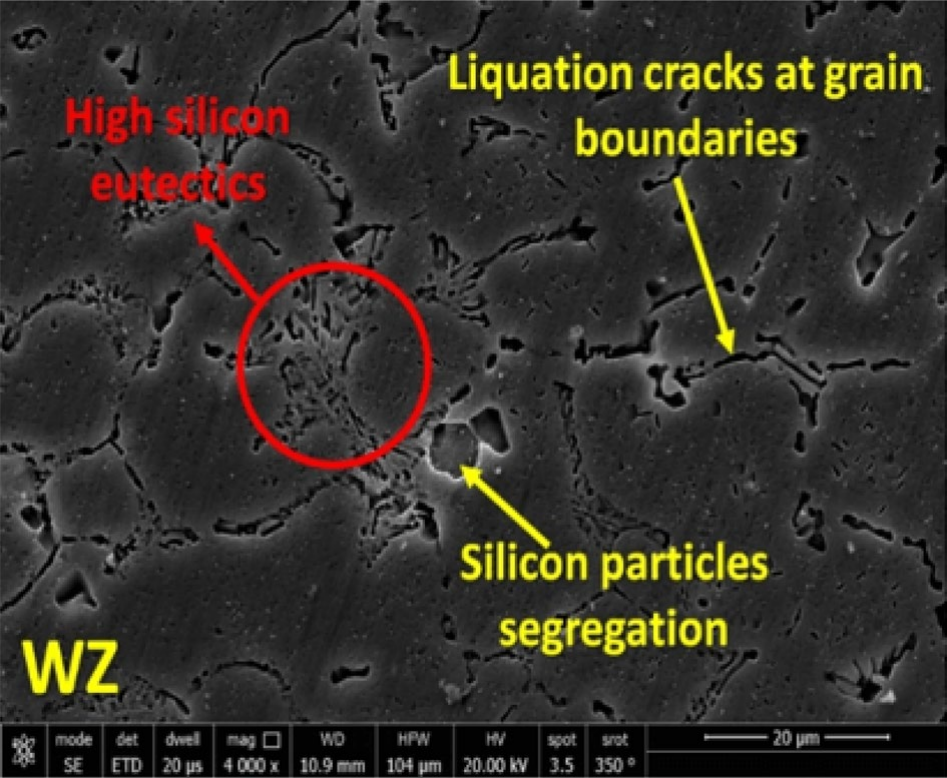
SEM photomicrograph of the weld zone of AA 5083 base alloys welded joints using ER 4043 filler metal showing the weld zone micro-cracks caused by liquation at grain boundaries and silicon particle segregation.

On the other hand, the microstructure of similar AA 5083 welded joints welded with ER 4043 filler reveals a micro shrinkage at grain boundaries of the WZ, as noted in Fig. 6a. Grain boundary liquation also occurred at the WZ-HAZ interface, as shown in Fig. 6b. Moreover, solidification cracks and macro shrinkages are observed at the WZ-BM root interface, as noted in Fig. 6c. Furthermore, the SEM observations indicate the presence of high silicon eutectics, particles of silicon segregation, and liquation cracks at the weld zone grain boundaries, as shown in Fig. 7.

The filler material selection is a critical factor affecting aluminum alloys' weld joint quality. In the present investigations, the ER 4043 filler metal was selected for joining different aluminum alloys AA 6082 and AA 5083 to obtain weld joints free from defects. However, solidification cracks and silicon segregation appeared in WZ during the solidification, similar to those previously noted^34–36^. Moreover, further explanation will be added in detail in the following paragraphs.

Redistribution of the solutes during the GTAW welding contributes to the appearance of silicon segregation, as noted previously^34^, and will be discussed in detail as follows. Based on the phase diagrams, the equilibrium distribution coefficient (k_0_) in the Al-Si alloys equals 0.13. The degree of segregation can be described according to Eq. (1) ^37^.

1 $$ {\text{C}}_{{\text{s}}} - {\text{ C}}_{{\text{l}}} = {\text{ C}}_{\alpha } \left( {{1 } - {\text{ k}}_{0} } \right) $$

C_s_ and C_l_ are the equilibrium concentration of solid and liquid, respectively, and C_α_ is the concentration of the main element (the Si, in the case of ER 4043 filler). The smaller the k_0_ (k_0_ < 1), the larger the segregation trend of Si. Si segregation appears near the grain boundaries in the nugget zone, as noted in Fig. 7. The Si tends to segregate to the liquid phase during solidification (k_0_ < 1), resulting a high silicon eutectics and silicon particles segregation at the grain boundaries. Based on the above microstructure investigations, it is clear that ER 4043 reduces the likelihood of hot cracks and improves weldability, but this is achieved if the base metal dilution is less than 70%^38^. Interestingly the calculation of the average % dilution of weldments in the current study was 24.76%. The obtained value was lower than that of 70%^38^ that confirm of the effectiveness of using ER 4043.

**Figure 8:**
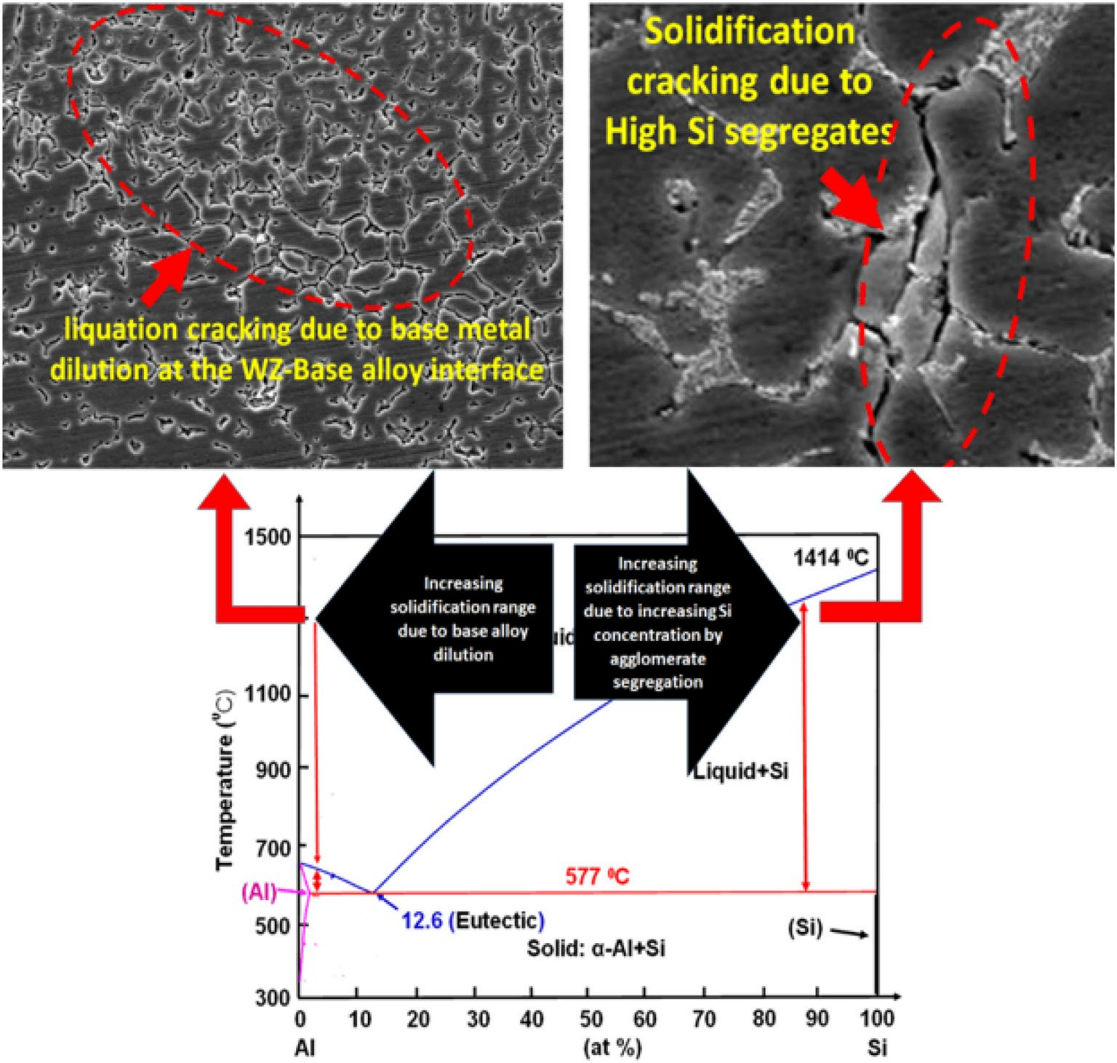
Al–Si phase diagram explaining the probable causes of increasing the solidification range that may lead to solidification cracking.

In the present work, ER 4043 needs a low melting point, so a lower heat input in the root is achieved. But the dilution effect is increased due to the lower quantity of ER 4043 filler in the root zone resulting in a high dilution of the base metal that leads to a shift of the chemical composition of the Al-Si alloys system towards the left-hand side (Fig. 8). Furthermore, it increases the probability of weld zone cracking due to rising of solidification range. Moreover, the segregation phenomena carried out during the WZ formation and solidification may lose the role of ER 4043 filler in improving weldability and preventing cracks by narrowing the freezing range, as shown in Fig. 8.

The cracking at the WZ of the ER 4043 filler weldment may be due to the increase of Si concentration by segregation at the mushy zones and increasing of solidification range. Two opposite effects occur due to the presence of silicon and magnesium. Increasing silicon concentration increases the chance of solidification cracking. On the other hand, increasing Mg concentration will reduce the probability of solidification crack formation^39^.

Moreover, the solidification cracks at the WZ-Base alloy interface may result from the formation of low-melting intermetallics such as Mg_2_Si precipitates. On the other hand, using ER 4043 at the root of the joints increase the solidification range. Consequently, the Si content in the fused weld pool depleted due to high base metal dilution, where the root gap is narrower than the upper part of the joint, as shown in Fig. 8. Therefore, using ER 4043 as filler in welding AA 6082 and AA 5083 aluminum alloys similar joints are not recommended.

##### Similar welding joints using ER 5356 filler

**Figure 9:**
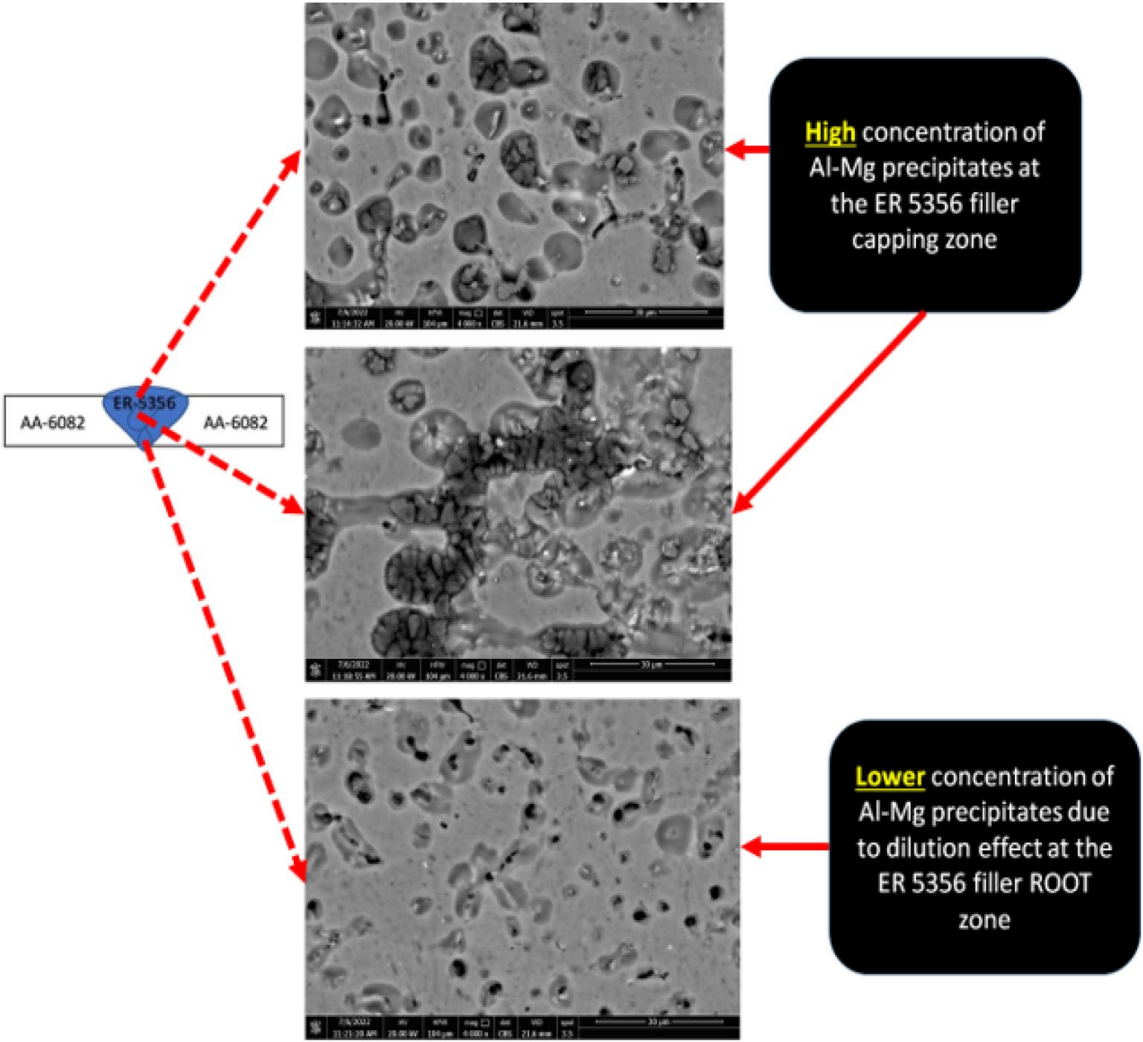
SEM photomicrograph of AA 6082 welded joint using ER 5356 filler rod and GTAW process.

**Figure 10:**
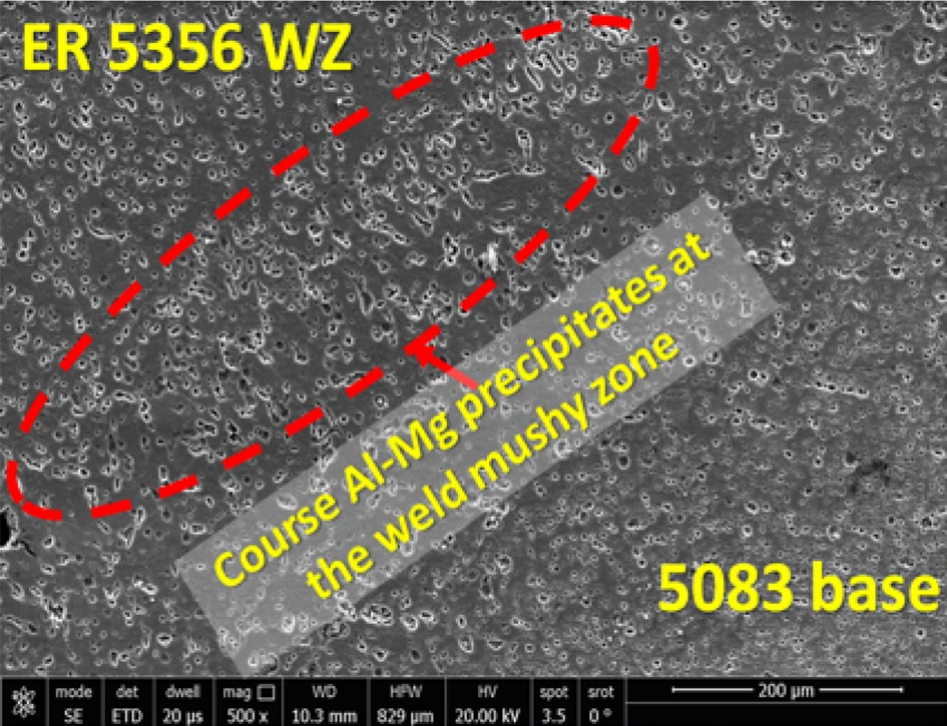
SEM photomicrograph of AA 5083 welded joint using ER 5356 filler rod and GTAW process.

In order to investigate the effect of the filler material on the quality of AA 6082 and AA 5083 similar welding joints, the ER 5356 filler was applied. The SEM photomicrographs of the upper cap, middle cap, and root zones of the AA 6082 weld similar joint welded using ER 5356 filler are shown in Fig. 9. It can be noted that the AlMg precipitates concentration at the upper and middle capping zones is higher than that in the root of the joint. This observation can be attributed to the dilution effect of AA 6082 base alloy in the root zone. On the other hand, the SEM micrograph of AA 5083 weld joint welded using ER 5356 is shown in Fig. 10. Course precipitates are formed in the mushy zone at the weld interface that resulted from the increasing solidification time (Fig. 10).

Interestingly no obvious solidification cracks or silicon particle segregation are noticed when using ER 5356, as shown in Figs. 9 and 10. Therefore the ER 5356 can effectively produce high-quality similar AA 6082 and AA 5083 weld joints relative to the ER 4043. However, the ER 5356 welding provided higher weld joint strength, hardness, and ductility than ER 4043^40^. However, using ER 4043 also has different advantages, such as its service temperatures above 340 K and a higher rating for weldability with a slightly lower crack sensitivity. Furthermore, smoother surfaces, less spatter, and smu can be obtained by using ER 4043. Therefore, further investigation about the effectiveness of using a mix between both fillers is still required through the dissimilar welding of AA 6082 and AA 5083, as shown in the following section.

#### Welding of dissimilar AA 6082 and AA5083 alloys joints

##### Dissimilar welding using ER 4043 filler

**Figure 11:**
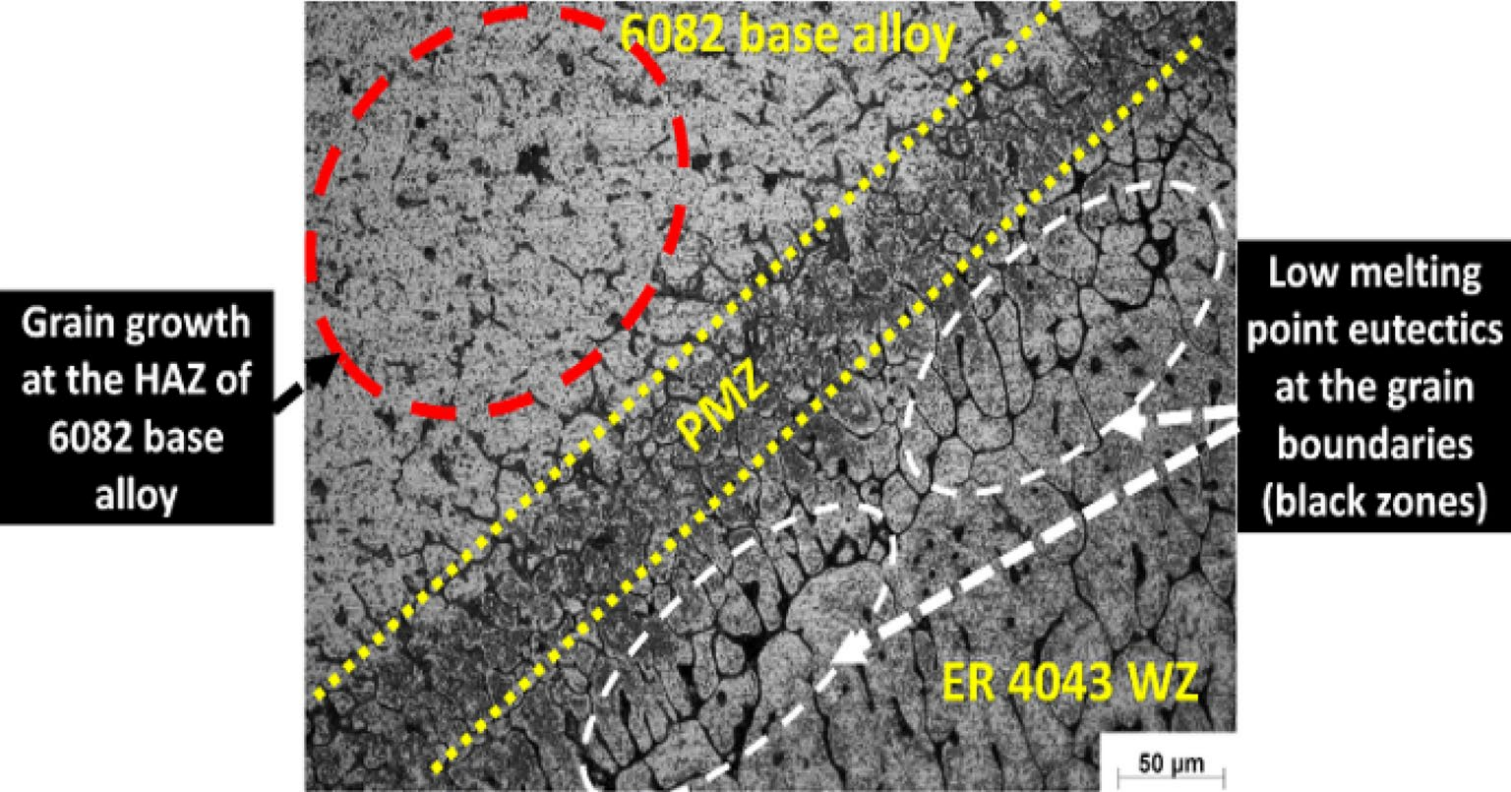
Optical micrograph of the boundary zone between AA 6082 base alloy and the weld zone (WZ) using ER 4043 filler rod.

**Figure 12:**
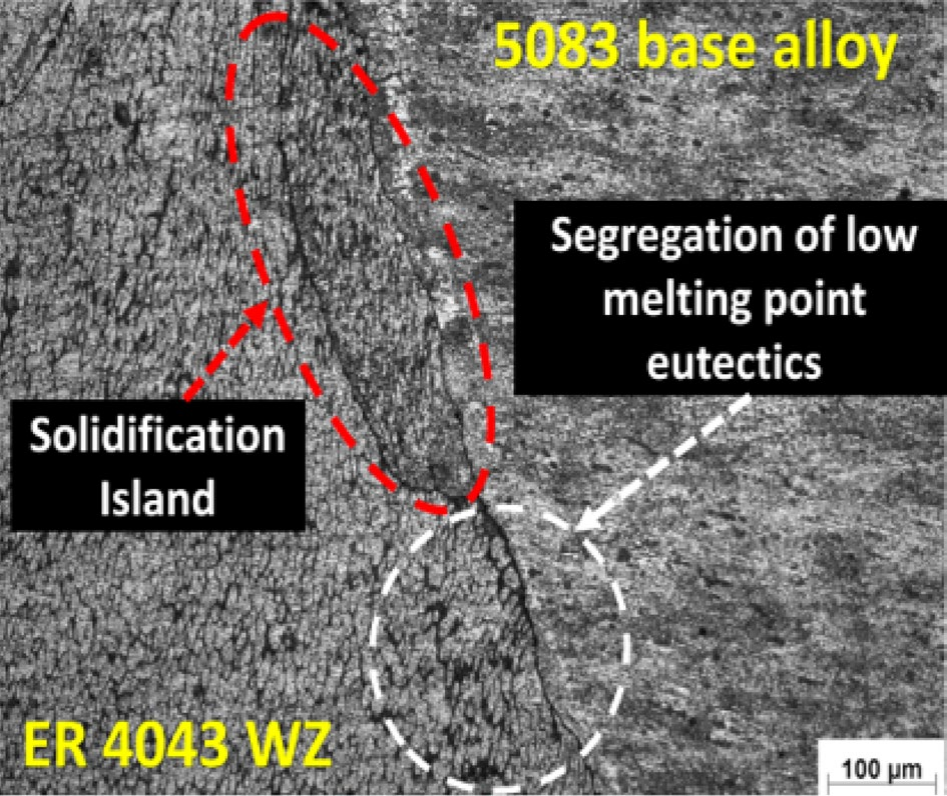
Optical micrograph of the boundary zone between AA 5083 base alloy and the weld zone (WZ) using ER 4043 filler rod.

The microstructure observation of the dissimilar AA 5083–AA 6082 welded joint welded using ER 4043 in WZ and HAZ for both sides of the joint is shown in Figs. 11 and 12. A good merging between the weld zone and AA 6082 base alloy is observed (Fig. 11). It is also observed that the WZ is enriched with the AlSi eutectic at the grain boundaries at the boundary zone close to the AA 6082 base alloy. Also, observed grain growth occurs at the heat-affected one of AA 6082 alloy.

On the other hand, a solidification island surrounded by hair liquation crack is observed at the boundary between AA 5083 base alloy and ER 4043 filler weld zone. This observation is due to the presence of the enrichment of Mg_2_Si brittle precipitates at this zone (Fig. 12). The difference in chemical composition between the ER 4043 and AA 5083 alloy leads to different solidification times, which contributes to the formation of a hot spot at the end of solidification in the weld zone (richen with ER 4043). Moreover, investigation of the heat-affected zone reveals a grain coarsening for AA 5083 alloy side, as shown in Fig. 12. The disbanding noted in the WZ of the AA 5083 side interface represented by the solidification island can attribute to the variation in the chemical composition between the two zones.

The effect of using ER 4043 on the phase formation for AA 6060 fusion welding was also noted by Coniglio et al.^41^. The ER 4043 enhances the weld quality and decreases the chance of solidification crack formation due to high Si content. Similar to that noted in the current study. On the other hand, the formation of cracks at the interface between the weld zone in the AA 5083 alloy side can be attributed to the dilution effect that results from the mixing between the filler and the base alloys, leading to the increase of the solidification range and so the formation of solidification cracks^42,43^. Fe also plays a significant role in aluminum solidification due to its strong tendency to partition (equilibrium partition ratio: k = 0.03). While ordinarily, Fe presents as an impurity in small amounts around 0.22 and 0.38 wt. Fe in AA 6082 and AA 5083, as indicated in Table 1. At the same time, the iron content is 0.8 wt.% in the case of ER 4043 filler. Iron forms intermetallic compounds with aluminum and silicon, affecting the solidification sequence^44,45^. Phases normally expected when casting an Al–Mg–Si–Fe quaternary alloy like alloy AA 6082 include ß-Al_5_FeSi, α-Al_8_Fe_2_Si, and π-Al_8_FeMg_3_Si_6_ phases in addition to Mg_2_Si and Si^46^.

**Figure 13:**
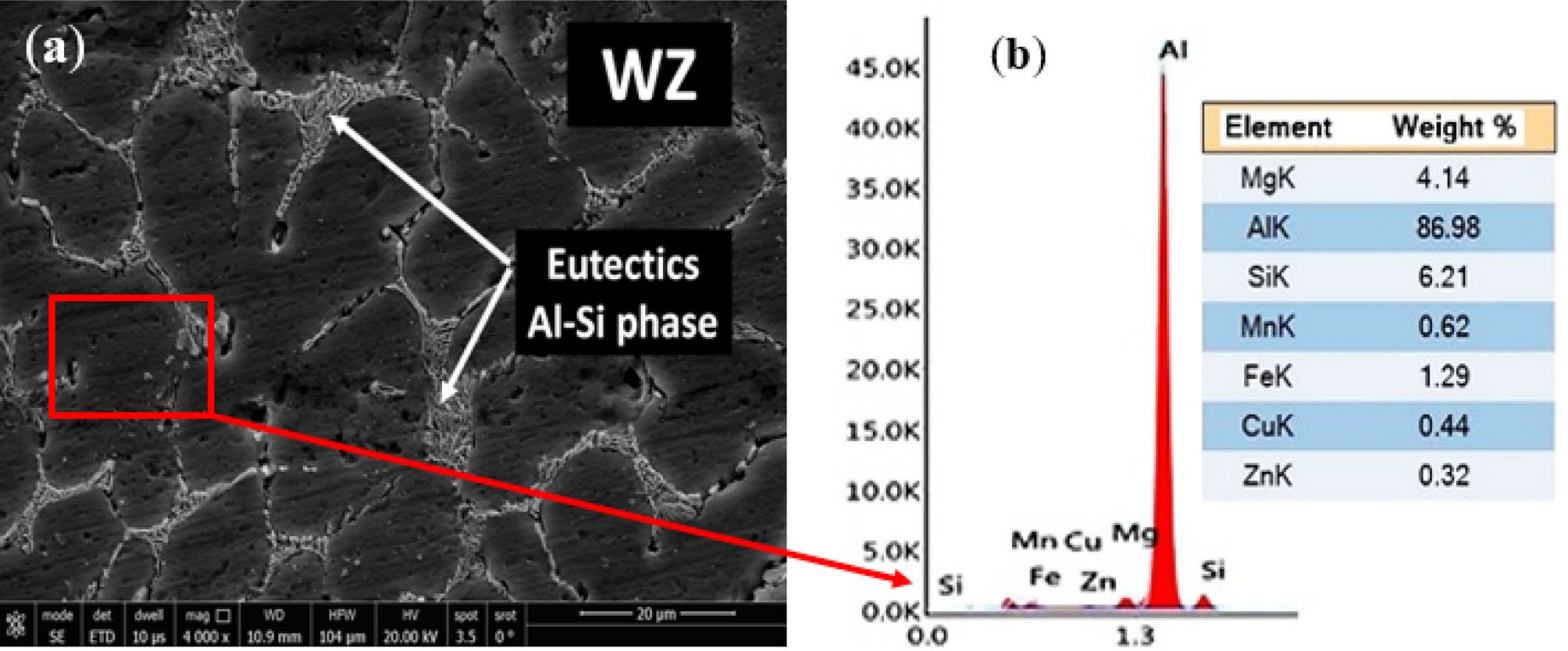
SEM of the dissimilar welded AA 5083 and AA 6082 aluminum joint using ER 4043 filler: (**a**) the weld zone (WZ) and (**b**) the EDS area analysis of the of AA 6082 alloy side.

The SEM photomicrograph of the dissimilar welded AA 5083 and AA 6082 aluminum joint using ER 4043 of the weld zone WZ of AA 6082 alloy side is shown in Fig. 13a. The EDS area analysis of the WZ in the dissimilar welding using ER 4043 filler in the AA 6082 alloy side shown in Fig. 13b indicates the presence of the elements required to form the mentioned compounds. Interestingly, the percent of the Fe noted was near from that of sum of that in both ER 4043 filler and AA 6082 alloy, that confirms the obtained results.

#### Dissimilar welding using multiple fillers ER 4043 filler rod at the root zone and ER 4356 rod at the capping zone

**Figure 14:**
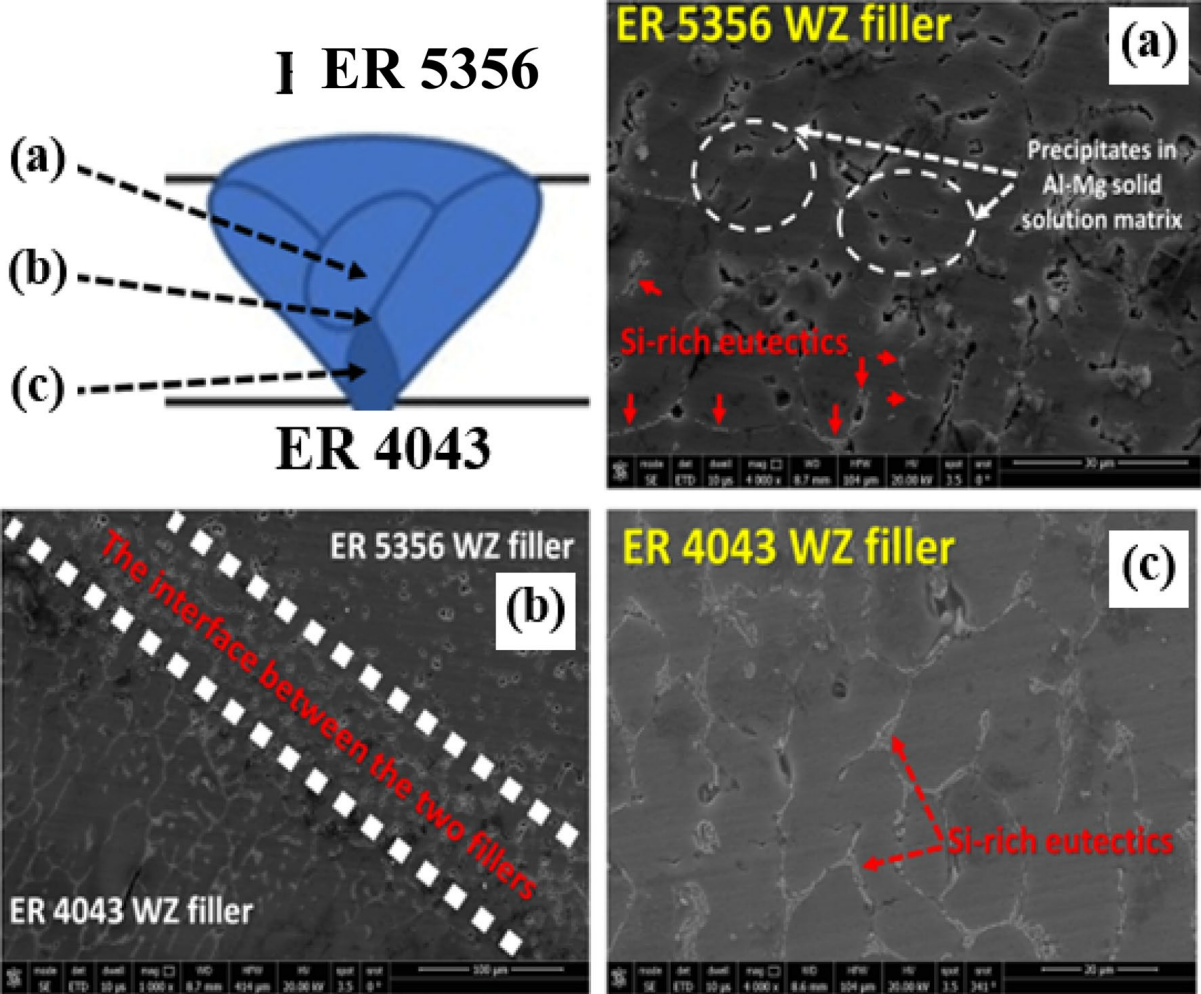
SEM photomicrograph details for the dissimilar AA 5083–AA 6082 welded joint using multiple fillers ER 4043 (root zone)–ER 5356 (capping zone) where: (**a**) the capping zone using ER 5356 filler, (**b**) the boundary between the two fillers solidified weld pool and (**c**) the root zone using ER 4043 filler.

The defects noted during the dissimilar welding of the AA 5083–AA 6082 alloys using ER 4043 filler motivated the use of a new technique in the dissimilar welding. The effect of the combination of the two fillers ER 4043 and ER 4356 was performed, as shown in Fig. 14, with the application of ER 4043 at the root zone and ER 4356 at the capping zone. Interestingly a defect-free joint is obtained when using multiple filler techniques for welding AA 5083–AA 6082 dissimilar joint (Fig. 14). So, the multiple filler technique is a good approach for weld zone chemical composition modification. Using multiple fillers controls and redistributes solute concentrations in the Al alloys welded joints. Pickin et al. discusses controlling weld zone composition for high-strength aluminum alloys using the tandem process^47^. It is concluded that using binary system fillers increases the probability of crack formation in the weld nugget.

In contrast, using ternary system fillers produces a crack-free weld joint. Using a mix of fillers (multiple fillers) contributes to forming a ternary alloy system in the weld nugget and lowers the chance for solidification crack formation. Using multiple filler techniques for welding AA 5083—AA 6082 dissimilar materials by ER 4043 at the root zone and ER 4356 not only contributes to producing high-quality weld joints but also reduces the overall cost of the weld joint. The lower cost of the ER 4043 relative to that of ER 4356 can reach half of that of ER 4356, producing cheaper weld joints of the dissimilar alloys AA 5083—AA 6082 with high quality.

## Corrosion properties investigation

### Corrosion of similar welding joints

#### Corrosion of similar welding joints using ER 4043 filler

**Figure 15:**
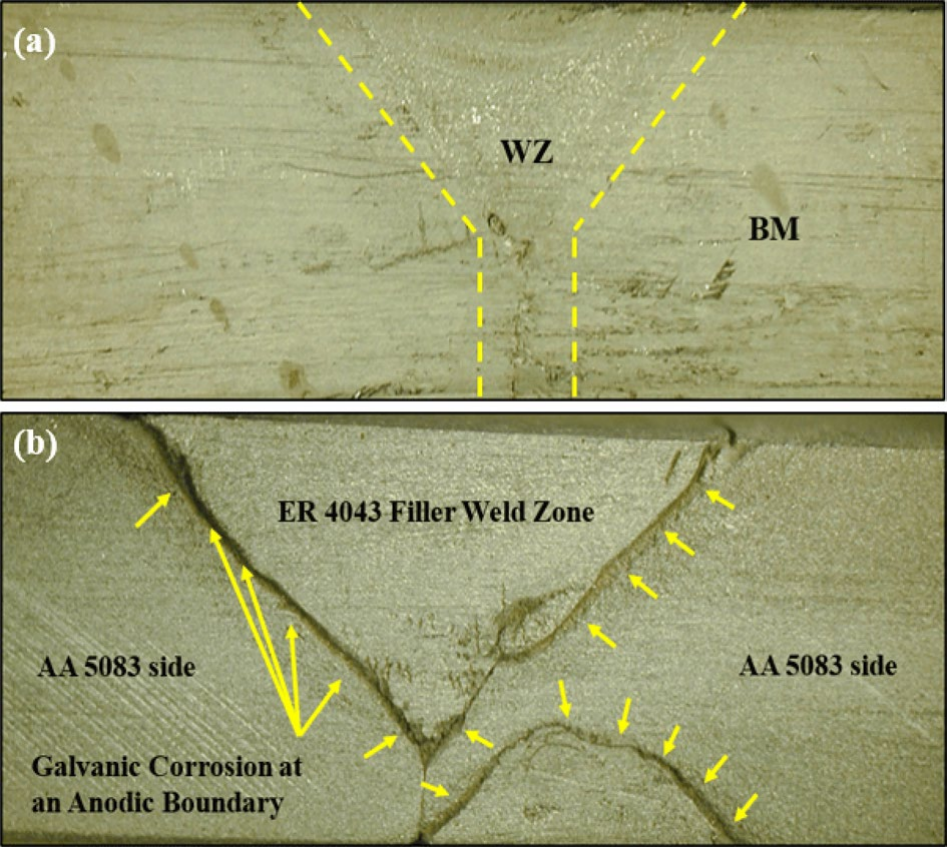
Macrograph of similar welded joints after corrosion immersion test: (**a**) welded joint of AA 6082 base alloy using ER 4043 filler rod and (**b**) welded joint of AA 5083 base alloy using ER 4043 filler rod.

The corrosion potentials in aluminum alloys depend mainly on the types of intermetallic that form during the production and processing of aluminum alloys^48,49^. The electrochemical behavior of aluminum alloys is affected by the alloying elements. Therefore, each Al-alloy has its electrochemical potential depending on its chemical composition. In the case of aluminum welded joints, the situation is severe and complex from the corrosion standpoint of view. In the present work, the immersion test of the welded joints reveals no galvanic attack between ER 4043 filler and AA 6082 base alloy, as shown in Fig. 15a. Whereas galvanic corrosion at the interface between the ER 4043 filler and AA 5083 base alloy is clear, as observed in Fig. 15b.

**Figure 16:**
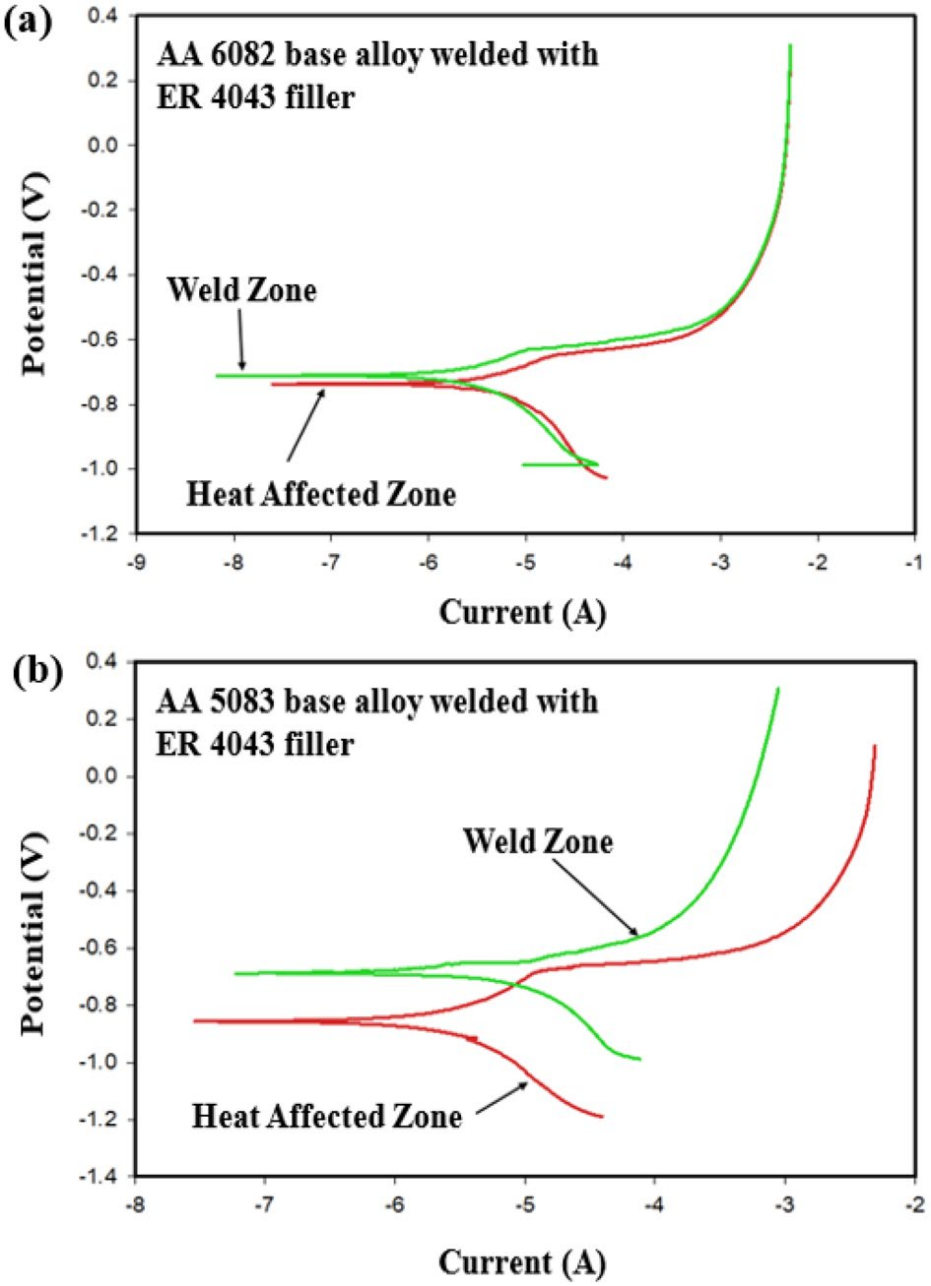
Results of electrochemical corrosion test for similar welded joint welded with ER 4043 filler: (**a**) AA 6082 base alloy and (**b**) AA 5083 base alloy.

Potentiodynamic polarization curves for AA 6082 base /ER 4043 and AA 5083/ER 4043 similar welded joints in 3.5% NaCl are shown in Fig. 16a, b respectively. The polarization plots indicate a narrow corrosion potential difference between the nugget zone and the WZ-HAZ interface zone for AA 6082/ER 4043 filler welded joint. This observation is due to the chemical behavior similarity between the weld zone and the adjacent parent alloy E_corr_ for nugget zone ~ −0.7 V vs. SCE (Standard Calomel Electode) and E_corr_ for WZ-HAZ interface ~ −0.72 V vs. SCE. On the other hand, more positive corrosion potential was noted for the ER 4043 weld zone compared with WZ-HAZ interface for AA 5083/ER 4043 filler welded joint. Where the WZ-HAZ interface is more anodic (E_corr_ ~ −0.83 V vs. SCE) compared with the adjacent nugget zone (E_corr_ ~ −0.68 V vs. SCE) as shown in Fig. 16b, this explains the galvanic attack of the WZ-HAZ interface macro section shown in Fig. 15b.

#### Corrosion of similar welding joints using ER 5356 filler

**Figure 17:**
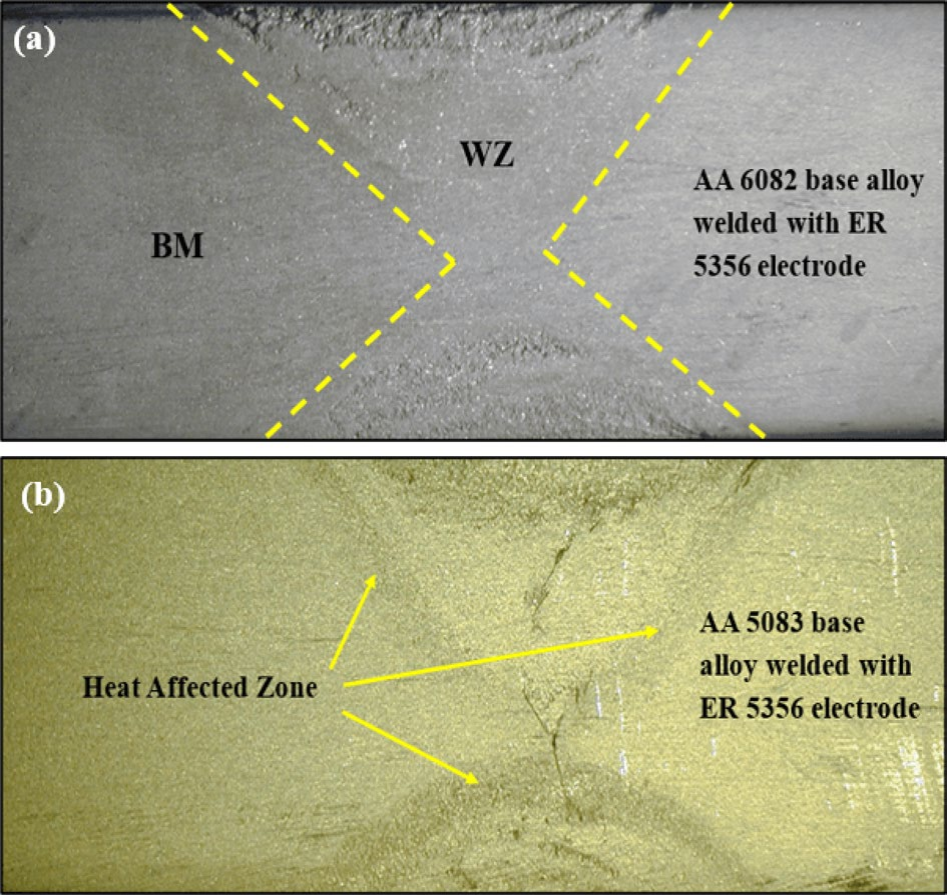
Macrograph of similar welded joints after corrosion immersion test: (**a**) welded joint of AA 6082 base alloy using ER 5356 filler rod and (**b**) welded joint of AA 5083 base alloy using ER 5356 filler rod.

**Figure 18:**
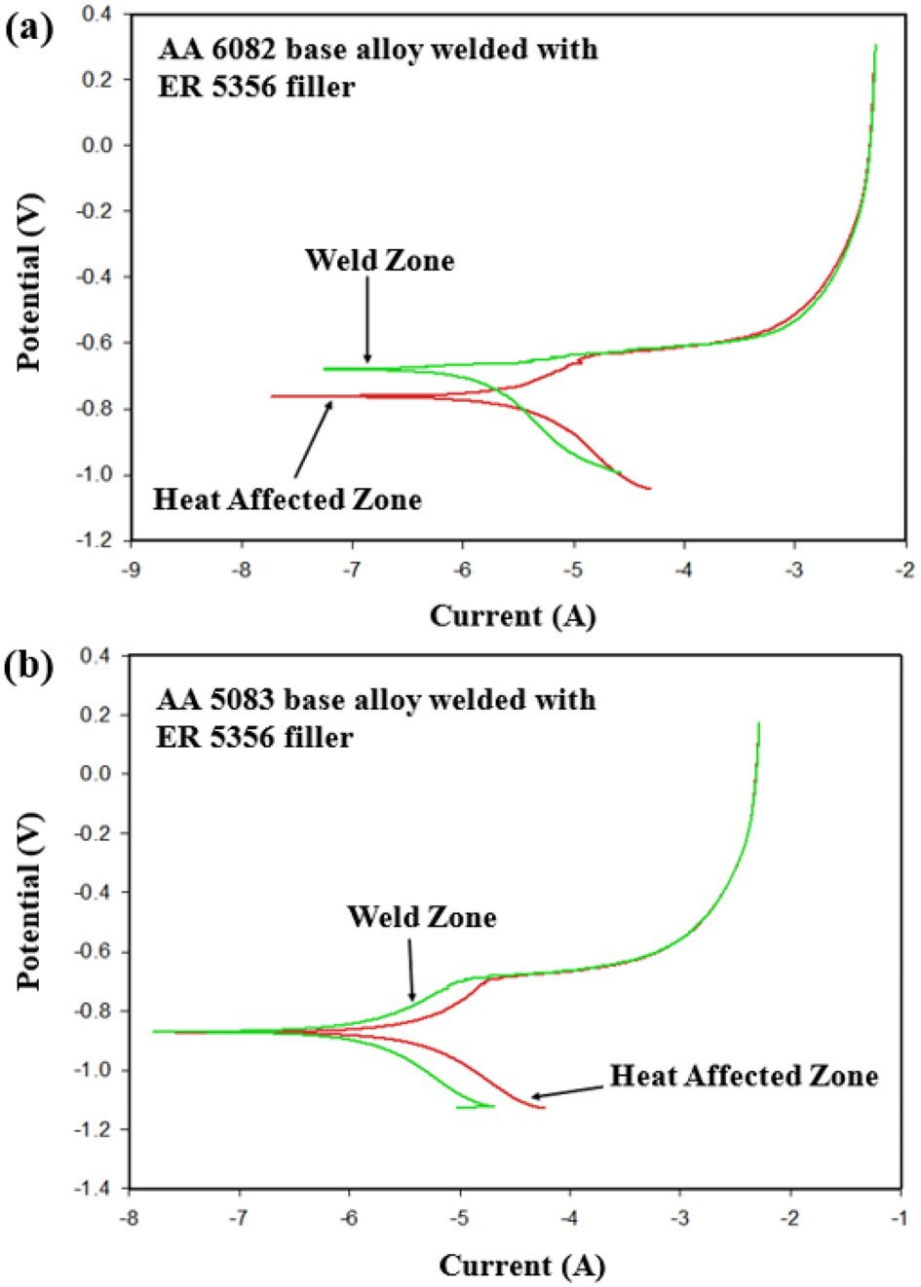
Results of electrochemical corrosion test for similar welded joint welded with ER 5356 filler: (**a**) AA 6082 base alloy and (**b**) AA 5083 base alloy.

In the similar welding joints welded using ER 5356, the immersion test of the welded joints reveals no galvanic attack, as observed in Fig. 17a,b. Moreover, polarization plots indicate a very narrow corrosion potential difference between the nugget zone and the WZ-HAZ interface zone for AA 6082/ER 5356 filler welded joint which means the chemical behavior similarity between the weld zone and the adjacent parent alloy E_corr_ for nugget zone ~ −0.69 V vs. SCE and E_corr_ for WZ-HAZ interface ~ −0.75 V vs. SCE, as indicated in Fig. 18a. On the other hand, it was observed that the corrosion potential values for both regions that equal E_corr_ ~ −0.88 V vs. SCE for both the weld zone and WZ-HAZ interface in the AA 5083/ER 5356 filler, as shown in Fig. 18b.

#### Corrosion of dissimilar welding joints

**Figure 19:**
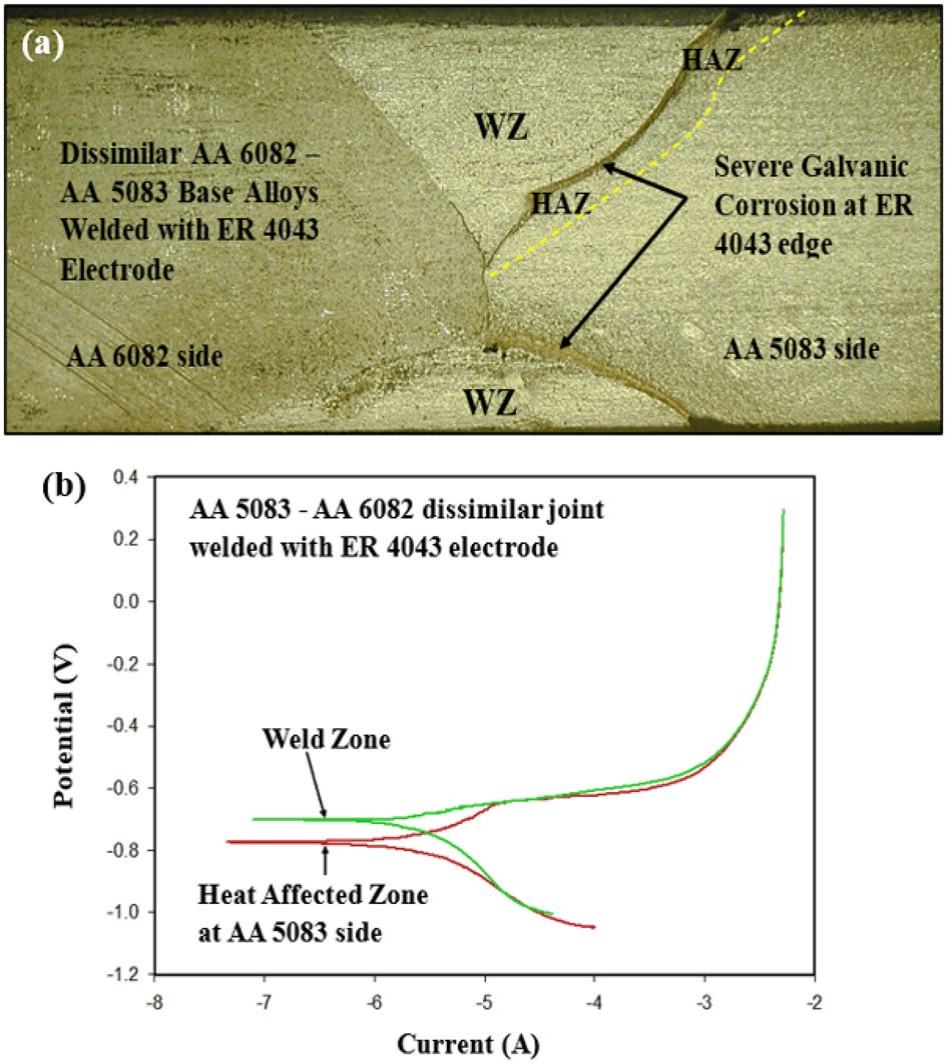
(**a**) Macrograph of dissimilar AA 5083–AA 6082 welded joints welded with ER 4043 filler rod after corrosion immersion test and (**b**) Results of electrochemical corrosion test for dissimilar AA 5083 and AA 6082 welded joint using single filler of ER 4043.

The results of the corrosion immersion test of dissimilar AA 6082–AA 5083 joints welded with ER 4043 filler rod are shown in Fig. 19a. A galvanic corrosion results in the vicinity of AA 5083 base alloy side, whereas a little attack is observed near the AA 6082 side. The attack is found in the WZ-HAZ interface indicating the chemical behavior dissimilarity between the WZ, and HAZ resulted from the difference in chemical composition between the regions.

The polarization plots (Fig. 19b) indicate a relatively small corrosion potential difference between the nugget zone and the WZ-HAZ interface zone for AA 5083/ER 4043 filler welded joint near the AA 5083 base alloy. This observation is due to the chemical behavior dissimilarity between the weld zone and the adjacent parent alloy E_corr_ for nugget zone ~ −0.7 V vs. SCE and E_corr_ for WZ-HAZ interface ~ −0.78 V vs. SCE (Fig. 19b). An important notice that the potential difference between WZ (or ER 4043 nugget) and the WZ-HAZ interface of AA 5083 similar joint (Fig. 15b) was ~ 0.15 V, whereas the potential difference between WZ (or ER 4043 nugget) and the WZ-HAZ interface of AA 5083 dissimilar joint (Fig. 19b) was ~ 0.08 V. This indicates that the using of dissimilar joints may enhance the corrosion resistance due to the modification of nugget chemical composition.

**Figure 20:**
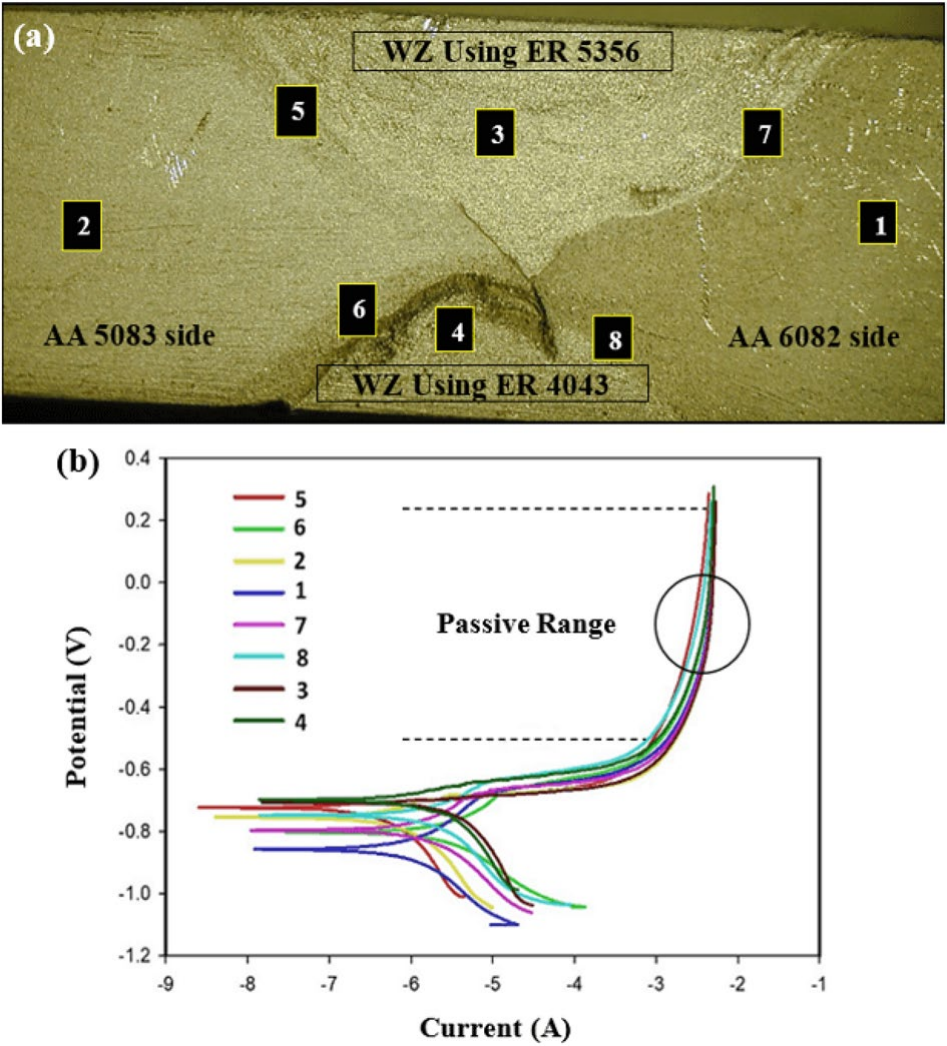
(**a**) Macrograph of dissimilar AA 5083–AA 6082 welded joints welded with ER 4043 filler rod in the root and ER 5356 filler in the cap after corrosion immersion test and (**b**) Results of electrochemical corrosion test for dissimilar AA 5083–AA 6082 welded joints welded with ER 4043 filler rod in the root and ER 5356 filler in the cap.

The AA 6082–AA 5083 dissimilar joint welded with ER 4043 filler rod in the root and ER 5356 in the cap after the immersion test is shown in Fig. 20a. A galvanic corrosion for the ER 4043 nugget interface resulted in the vicinity of AA 5083 base alloy side, whereas no attack was observed near the AA 6082 side. The attack is found in the WZ-HAZ interface indicating the chemical behavior dissimilarity between the WZ, and HAZ resulted from the difference in chemical composition between the regions.

**Figure 21:**
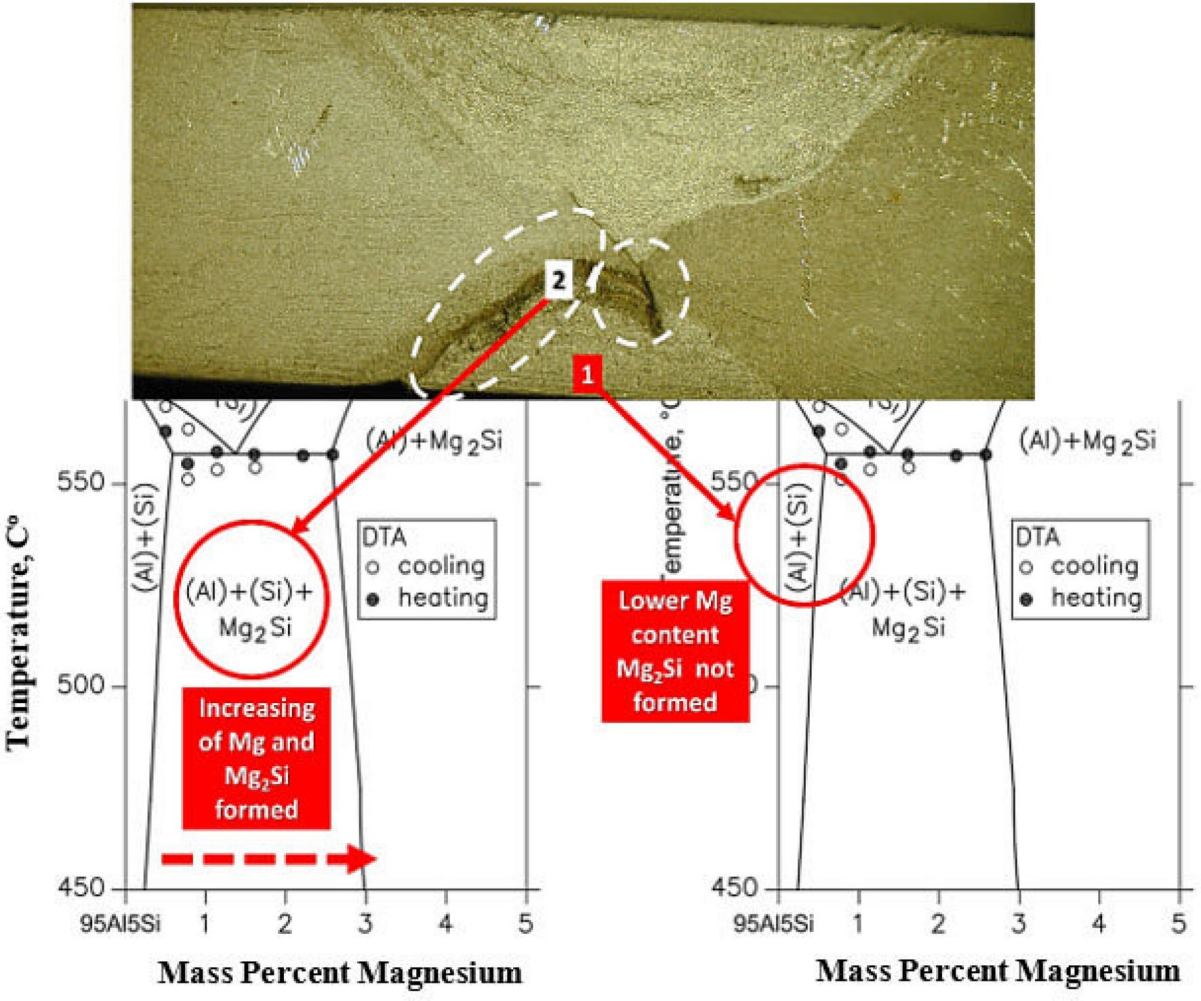
The relation between the corrosion attacks of the ER 4043–AA 5083 and ER 4043–ER 5356 interfaces and the Al–Mg–Si phase diagram^53^.

The polarization plots (Fig. 20b) indicate a relatively small corrosion potential difference between the ER 4043 and ER 5356 nugget zones and the WZ-HAZ interface zones adjacent t to both base alloys AA 6082 and AA 5083. From the literature^50^, aluminum–silicon alloys generally have good corrosion resistance unless they are in contact with more noble alloys (e.g., 5000 series) like the present work where ER 4043 filler in the weld zone comes in contact with AA 5083 base alloy and ER 5356 filler in case of dissimilar fillers (Fig. 20a,b), leading to the formation of magnesium silicide, and magnesium silicide is highly anodic compared with all parts of the welded joint^51,52^. Relating the corrosion attacks of the ER 4043—AA 5083 and ER 4043—ER 5356 interfaces to the Al–Mg–Si phase diagram^53^ shown in Fig. 21. At lower Mg content, no Mg_2_Si precipitates formed, whereas increasing Mg contents (at the WZ-HAZ interfaces) where the dilution of Al–Mg materials predominates Mg_2_Si precipitates is formed as seen in the phase diagram at higher Mg content.

## Conclusions

Through the current research, it was concluded the following:

- It is recommended to use ER 5356 fillers in welding both AA 6082 and AA 5083 similar weld joints over using ER 4043 fillers that overcome the formation of solidification cracks due to improper solidification.
- The appearance of the solidification cracking while using the ER 4043 filler depends on shifting the composition of the Al-Si to the right of the phase diagram. As the solidification range increase, enhance the solidification cracks formation probability due to the increase in silicon content.
- Formation of solidification cracking at the root zone for ER 4043 filler caused by the base metal dilution and shifting the chemical composition to the left side of the Al-Si phase diagram. On the other hand, in the capping zone at the WZ-HAZ interface, cracking may be formed due to low melting point constituents.
- Using dissimilar fillers in welding AA 6082 and AA 5083 alloys enhances the microstructure with crack-free weldments.
- ER 5336 filler electrode is more favorable than ER 4043 filler electrode for dissimilar welding of AA 5083 and AA6082 alloys or individual welding of aluminum alloys. Moreover, no galvanic corrosion is observed between ER 4043 fillers and AA 6082 alloy.
- Dissimilar fillers enhance the corrosion resistance due to the change in the nugget zone's chemical composition and decrease the contrast in corrosion potentials between the different zones of weld joints.

## Data Availability

The datasets used and/or analyzed during the current study available from the corresponding author on reasonable request.
