# Author Correction: Effects of filler on the microstructure and corrosion of similar and dissimilar gas inert tungsten arc welding aluminum alloys joints

**DOI:** 10.1038/s41598-023-48041-4

**Published:** 2023-11-23

**Authors:** Elshafey Ahmed Gadallah, Mohamed Ibrahim Abd El Aal, Abdelkarim Yousif Mohamed, Hossam Hemdan El-Fahhar

**Affiliations:** 1 Mechanical Production Department, Faculty of Technology and Education, Suez University, Suez, 43527 Egypt; 2 Mechanical Engineering Department, College of Engineering at Wadi Addawaser, Prince Sattam Bin Abdulaziz University, 18734 Wadi Addawaser, Saudi Arabia; 3 Mechanical Design and Production Department, Faculty of Engineering, Zagazig University, Zagazig, 44519 Egypt

Correction to: *Scientific Reports* 10.1038/s41598-023-44421-y, published online 03 November 2023

In the original version of this Article, the Acknowledgements section has been removed due to an incorrect grant fund and partial duplication of the Funding section.

The original Article has been corrected.

